# Ultrastructural Aspects of Corneal Functional Recovery in Rats Following Intrastromal Keratocyte Injection

**DOI:** 10.1167/iovs.66.2.45

**Published:** 2025-02-18

**Authors:** Qian Ma, Andri K. Riau, Robert D. Young, James S. Bell, Olga Shebanova, Nicholas J. Terrill, Gary H. F. Yam, Evelina Han, Keith M. Meek, Jodhbir S. Mehta, Craig Boote

**Affiliations:** 1 Structural Biophysics Research Group, School of Optometry and Vision Sciences, Cardiff University, Cardiff, United Kingdom; 2 Tissue Engineering and Cell Therapy Group, Singapore Eye Research Institute, Singapore, Singapore; 3 Ophthalmology and Visual Sciences Academic Clinical Programme, Duke-NUS Medical School, Singapore, Singapore; 4 Diamond Light Source, Harwell Science & Innovation Campus, Didcot, United Kingdom; 5 Department of Ophthalmology, University of Pittsburgh, Pittsburgh, Pennsylvania, United States; 6 Cornea and External Disease Department, Singapore National Eye Centre, Singapore, Singapore

**Keywords:** corneal stroma, collagen fibrils, proteoglycans, keratocytes

## Abstract

**Purpose:**

Donor tissue shortfalls and postsurgical complications are driving novel corneal tissue regeneration approaches. Corneal stromal keratocytes (CSKs) have shown promise in promoting corneal repair and restoring transparency. We investigated the impact of intrastromal CSK injection on corneal ultrastructure and proteoglycan (PG) distribution in a rat injury model.

**Methods:**

Rats were divided into four groups: normal (*n* = 12), injured (irregular phototherapeutic keratectomy centrally; *n* = 6), CSK (injured + human CSK intrastromal injection; *n* = 6), and PBS (injured + PBS injection; *n* = 6). Three weeks after treatment, corneas were examined by slit-lamp and optical coherence tomography. Corneal ultrastructure was analysed via small-angle x-ray scattering (collagen fibril diameter, interfibrillar spacing and matrix order), transmission electron microscopy with cuprolinic blue before and after chondroitinase digestion (CS/DS and KS PGs), and immunofluorescence staining (lumican and decorin).

**Results:**

Irregular phototherapeutic keratectomy caused corneal opacity and significantly disrupted stromal ultrastructure, characterized by increased haze density (*P* < 0.0001), change in central corneal thickness (*P* = 0.0005), and interfibrillar spacing (*P* < 0.0001), along with decreased fibril diameter (*P* < 0.0001), matrix order (*P* < 0.0001), CS/DS (*P* < 0.0001) and KS (*P* < 0.0001) PGs, lumican, and decorin. CSK injection recovered corneal clarity and native stromal ultrastructure, with haze density (*P* = 0.8086), change in central corneal thickness (*P* = 0.9503), fibril diameter (*P* = 0.1139), interfibrillar spacing (*P* = 0.5879), matrix order (*P* = 0.9999), CS/DS (*P* = 0.9969) and KS (*P* = 0.2877) PGs, lumican, and decorin returning to normal. In contrast, the PBS group exhibited similar corneal injury responses to the injured group.

**Conclusions:**

CSK injection resolved early stage corneal scarring by restoring stromal collagen arrangement and PG distribution, further endorsing its potential for treating corneal opacities.

Corneal transparency depends on the narrow, uniform diameter of type I collagen fibrils and their orderly arrangement within the corneal stroma.^1^^,^^2^ The precise organization of collagen fibrils is regulated in considerable part by small leucine-rich proteoglycans, consisting of a core protein and glycosaminoglycan (GAG) side-chains. Corneal proteoglycans (PGs) can be categorized into two main types: chondroitin sulfate/dermatan sulfate (CS/DS) PGs and keratan sulfate (KS) PGs, based on the specific types of GAG chains they contain.^3^ GAG chains exert distinct influences on the properties and functions of PGs.^4^ Research by Scott and Haigh^5^ has demonstrated that these two different types of PGs are located at distinct binding sites on collagen fibrils. CS/DS PGs are predominantly found at the “d” and “e” bands of collagen, whereas KS PGs are associated with the “a” and “c” bands. Beyond their role in maintaining corneal transparency through their involvement in collagen fibril growth and assembly of stromal lamellae, PGs also play regulatory roles in corneal wound healing by participating in biological processes, including cell migration and proliferation, cell adhesion, cell signaling, and inflammatory responses.^4^^,^^6^

The compositional and ultrastructural consequences of corneal injury and wound healing have been documented widely and include significant postinjury changes in stromal PG populations.^7^^–^^11^ Trauma, surgery, infection, and chemical burns disrupt the stromal lamellar ultrastructure, leading to increased light scattering, opacity, and vision impairment.^12^ Corneal opacities are a leading cause of blindness globally, with corneal injuries accounting for approximately 1.5 to 2.0 million new cases of unilateral blindness per year.^13^^,^^14^ Currently, corneal transplantation remains the primary treatment for persistent corneal opacity, but donor tissue shortages and suboptimal long-term outcomes that include graft rejection, infection, corneal endothelial failure, and glaucoma, can affect long-term outcomes.^15^ These challenges continue to motivate the development of alternative, minimally invasive therapeutic strategies.

Previous studies have emphasized the role of corneal stromal keratocytes (CSKs) in maintaining corneal transparency and their potential for stromal tissue regeneration.^16^^–^^18^ Originating from the neural crest, CSKs actively synthesize collagen and PGs to help maintain proper organization of the corneal stromal extracellular matrix.^19^ Moreover, upon injury, CSKs become activated and engage in complex physiological activities to repair corneal stromal wounds.^6^ We have previously demonstrated the therapeutic potential of injection of human CSKs to recover corneal transparency in rats with stromal injury induced by irregular phototherapeutic keratectomy (irrPTK).^16^^,^^20^ Herein, we further expanded on this work by assessing the impact of irrPTK injury on the corneal stromal ultrastructure and its subsequent recovery by CSK injection. We used small-angle x-ray scattering (SAXS) to quantify spatially resolved changes in collagen ultrastructural parameters across the cornea and used chondroitinase ABC digestion and cuprolinic blue critical electrolyte staining, combined with transmission electron microscopy (CB-TEM), to quantitatively assess the distribution of PG populations and visualize their associations with collagen.

## Methods

### Isolation and Propagation of Human Quiescent CSKs

Three pairs of research-grade human corneas, unsuitable for transplantation, were obtained from Saving Sight (Kansas City, MO, USA) (Table). The selection criteria were: age 52 years or younger, nondiabetic, death to processing time of 17 days or fewer, and no stromal haze in the central cornea. Corneas were preserved in Optisol-GS (Bausch + Lomb, Bridgewater, NJ, USA) at 4°C and transported to the laboratory. To cultivate quiescent CSKs, the central 8 mm of the corneas were trephined and processed for cell culture within 24 hours of receipt, as described previously, with a minor modification to the isolation protocol.^21^ Corneal stroma, with the epithelium and endothelium removed, were digested by 0.1% collagenase (Nordmark Pharm GmbH, Uetersen, Germany) and 0.1% bovine serum albumin (Sigma-Aldrich, St. Louis, MO) for 8 hours at 37°C. The isolated cells were propagated in activated form in CSK medium supplemented with 0.5% fetal bovine serum (Thermo Fisher Scientific, Waltham, MA, USA) on a collagen I–coated plate until passage 4. The CSK medium consisted of DMEM/F-12 (Thermo Fisher Scientific), 0.1 × insulin-transferrin-selenium (Thermo Fisher Scientific), 1 × nonessential amino acids (Thermo Fisher Scientific), 1 × vitamin solution (Thermo Fisher Scientific), 1 × antibiotic/antimycotic solution (Thermo Fisher Scientific), 1 × MEM essential amino acids (Thermo Fisher Scientific), 5 µg protein/mL of human amnion stromal extract, 10 ng/mL of insulin growth factor-1 (Thermo Fisher Scientific), 10 µM of ROCK inhibitor Y-27632 (Miltenyi Biotec, Bergisch Gladbach, Germany), and 0.5 mM of L-ascorbic 2-phosphate (Sigma-Aldrich). The activated cells were passaged at approximately 70% confluency using TryPLE express (Thermo Fisher Scientific). Media, reagents, and growth factors from the same production batch were used to minimize variability in the CSK culture outcomes. To generate quiescent CSKs, the cell culture was switched to a serum-free CSK medium for 14 days. All experiments were performed using cells at passage 3 or 4.

**Table.** Cornea Donor's Information

S/N	Age	Sex	Past Medical History	Cause of Death
1	46	Male	Hepatic encelopathy and alcohol abuse	End-stage liver disease
2	52	Male	Hypertension, hyperlipidemia, myocardial infarction, tobacco use, and hyperthyroidism	Sepsis
3	35	Male	Melanoma on back, tobacco and alcohol abuse	Subarachnoid hemorrhage

### Rat Corneal Injury Model and CSK Treatment

All animal procedures were conducted in accordance with the ARVO Statement for the Use of Animals in Ophthalmic and Vision Research approved by the Institutional Animal Care and Use Committee of SingHealth Duke-NUS (protocol no. 2019/SHS/1470). Eighteen Sprague-Dawley rats, aged 6 to 8 weeks, were used for the study. Intraperitoneal anesthesia was administered using a combination of xylazine (Troy Laboratories, Glendenning, Australia) and ketamine (Parnell Laboratories, Alexandria, Australia). Corneal wounds were induced by irrPTK as previously described.^16^^,^^22^ In brief, in one randomly selected eye of each rat, the corneal epithelium was removed up to approximately 0.5 mm from the limbus using a #64 surgical blade (BD, Franklin Lakes, NJ). The central 3mm area was then ablated using a Technolas 217z excimer laser (Bausch + Lomb), reaching a depth of 15 µm. A fine metal mesh was placed above the ablation zone after applying 50% of the laser pulse. The contralateral eye, which remained unwounded, served as the control (referred to as the normal group). After laser ablation, the rats received topical tobramycin (Tobrex; Alcon, Geneva, Switzerland) four times a day for 3 days. Seven days after injury, six of the wounded eyes were randomly assigned to the CSK group, which underwent intrastromal injection of quiescent CSKs by an experienced corneal surgeon (J.S.M.). After anesthesia, a stromal tunnel at the edge of the haze region in the anterior stroma was created with a 31G needle. Following that, 4 × 10^4^ cells in 2 µL of 1× PBS were injected through the tunnel using a 30G blunt needle attached to a Hamilton syringe (Hamilton Company, Reno, NV, USA). The chosen dosage was informed by our previous efficacy studies.^18^^,^^20^ Another six random wounded eyes served as sham controls and received intrastromal injection of 2 µL of 1× PBS without CSKs. The other wounded eyes were left untreated (referred to as the injured group). Rats in all four groups received tobramycin and dexamethasone eye drops (Tobradex; Alcon) four times a day for 7 days.

### Postoperative Ophthalmic Examination

All ophthalmic imaging was performed on day 21 after cell injection. Previous work characterizing the rodent irrPTK corneal injury model has established the peak of inflammatory cell density and stromal haze to be at 2 to 4 weeks.^23^ Thus, the 21-day period was chosen to assess the therapeutic effect of CSK treatment at the likely peak of stromal ultrastructural disruption. Slit-lamp photographs were taken using a Zoom Slit Lamp NS-2D (Righton, Tokyo, Japan). A masked grader (A.K.R.) was assigned to score the extent of haze using the Fantes method.^24^ Visualization of the corneal cross-section was performed using an Optovue anterior segment-optical coherence tomography (AS-OCT) device (Visionix, North Lombard, IL, USA). The central corneal thickness was measured with the built-in software in the AS-OCT. The change in central corneal thickness was obtained by the ratio of the difference between the corneal thicknesses on day 21 after injection and before preoperation to the preoperative central corneal thickness. In addition, the haze density in the center 3 mm of the cornea was evaluated with Fiji software version 2.9.0 (National Institutes of Health, Bethesda, MD, USA). Thereafter, the rats were sacrificed by intraperitoneal injection of overdosed pentobarbital (Jurox, Rutherford, Australia) under anesthesia. After enucleation, the corneas were dissected, wrapped in Saran Wrap (SC Johnson, Racine, WI, USA), and snap frozen in liquid nitrogen-cooled isopentane (Sigma-Aldrich) before storage at −80°C for SAXS and CB-TEM analysis.

### SAXS Protocol

**Figure 1.**
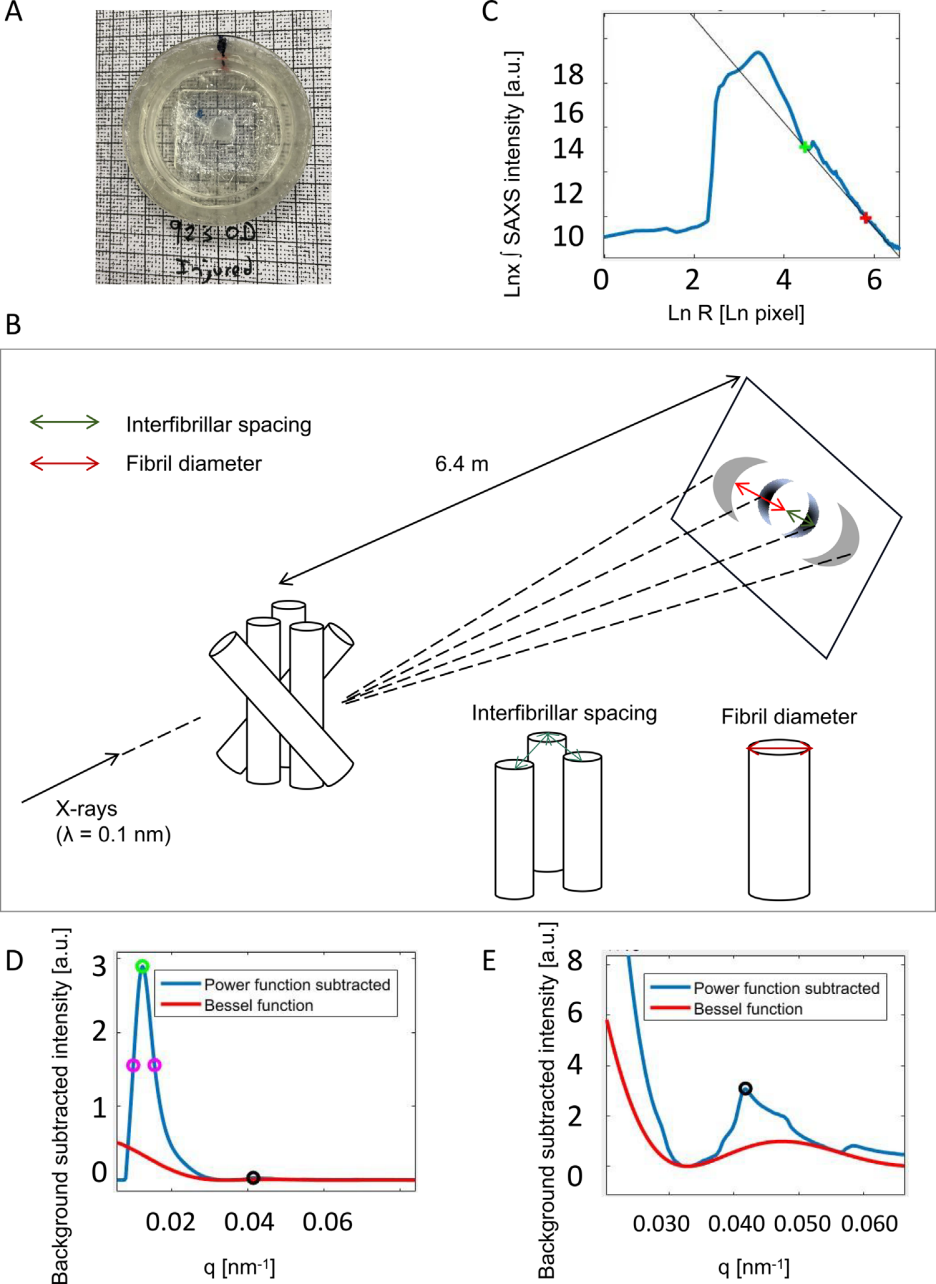
Collagen SAXS data collection and analysis. (**A**) Rat cornea sealed in x-ray sample cell. (**B**) Schematic of SAXS method to quantify fibrillar collagen parameters. (**C**) Logarithmic background noise (*solid black line*) subtraction from sample signal. (**D**) Resulting background-subtracted equatorial SAXS collagen signal (*blue*) with software-detected interfibrillar peak position marked by *green circle* and detected *purple circles* denoting the peak width at half height. The three circle coordinates were used to determine the height to half height width ratio of the interfibrillar peak as measure of the collagen matrix order. The *red line* shows the fitted model of the equatorial cylinder transform (based on a first order Bessel function)—the scatter from an isolated fibril. The *black hollow circle* corresponds with the third meridional reflection arising from the collagen axial periodicity (resolvable from the equatorial signal in expanded view in **E**). (**E**) Expanded view of the first subsidiary maximum peak region of the cylinder transform (*red*), showing model fitting to SAXS equatorial data (*blue*). The fitted peak position provides a measurement of the average fibril diameter. The detected meridional collagen axial D-period third order peak (black circle) is resolvable from the equatorial signal.

Frozen corneal specimens were transported to the Diamond Light Source national synchrotron (Didcot, UK) for SAXS data collection. SAXS was performed on noncrystalline Beamline I22^25^ following procedures described previously.^26^ X-ray–sensitive film was used to determine the x-ray beam position, and powdered silver behenate with a crystal structure dimension of 5.838 nm served as a calibrant. The wrapped corneas were thawed and placed in a sealed Perspex (Lucite Group Ltd., Southampton, UK) cell with a Mylar (DuPont-Teijin, Middlesbrough, UK) window (Fig. 1A). X-rays with a wavelength of 0.1 nm were directed parallel to the corneal optical axis, and exposures were recorded across the 3.5 mm × 3.5 mm central corneal region at intervals of 0.5 mm (horizontal) × 0.5 mm (vertical). SAXS patterns were recorded by a detector positioned 6.4 m behind the specimen (Fig. 1B).

SAXS data analysis was conducted using a combination of bespoke software tools: data analysis workbench (DAWN)^27^ and SAXS4COLL.^28^ Raw data, in Nexus fill format, were converted to TIF image format for subsequent analysis. The powder calibration image was first used to identify the SAXS pattern center and calibrate the radial positions of diffraction peaks on the detector. Data lost at the modular detector gaps were recovered by taking advantage of the centro-symmetry of the fiber pattern. Background scattering originating from tissue and specimen cell elements other than fibrillar collagen was then fitted and removed (Fig. 1C). On the radial intensity plot, equatorial reflections corresponding to the interfibrillar spacing (Gaussian-like interference function) and fibril diameter (cylinder transform based on first-order Bessel function)^29^ were identified and measured (Figs. 1D, E). Collagen matrix order was determined by calculating the height of the interference function peak divided by its width at half height. For each group, the average structural parameter contour maps were created by calculating the mean values of corresponding points across all specimens within that group. The 13 values from the central 2 mm × 2 mm region of all corneas in each group were used for statistical analysis.

### CB-TEM and Enzyme Digestion Protocols

**Figure 2.**
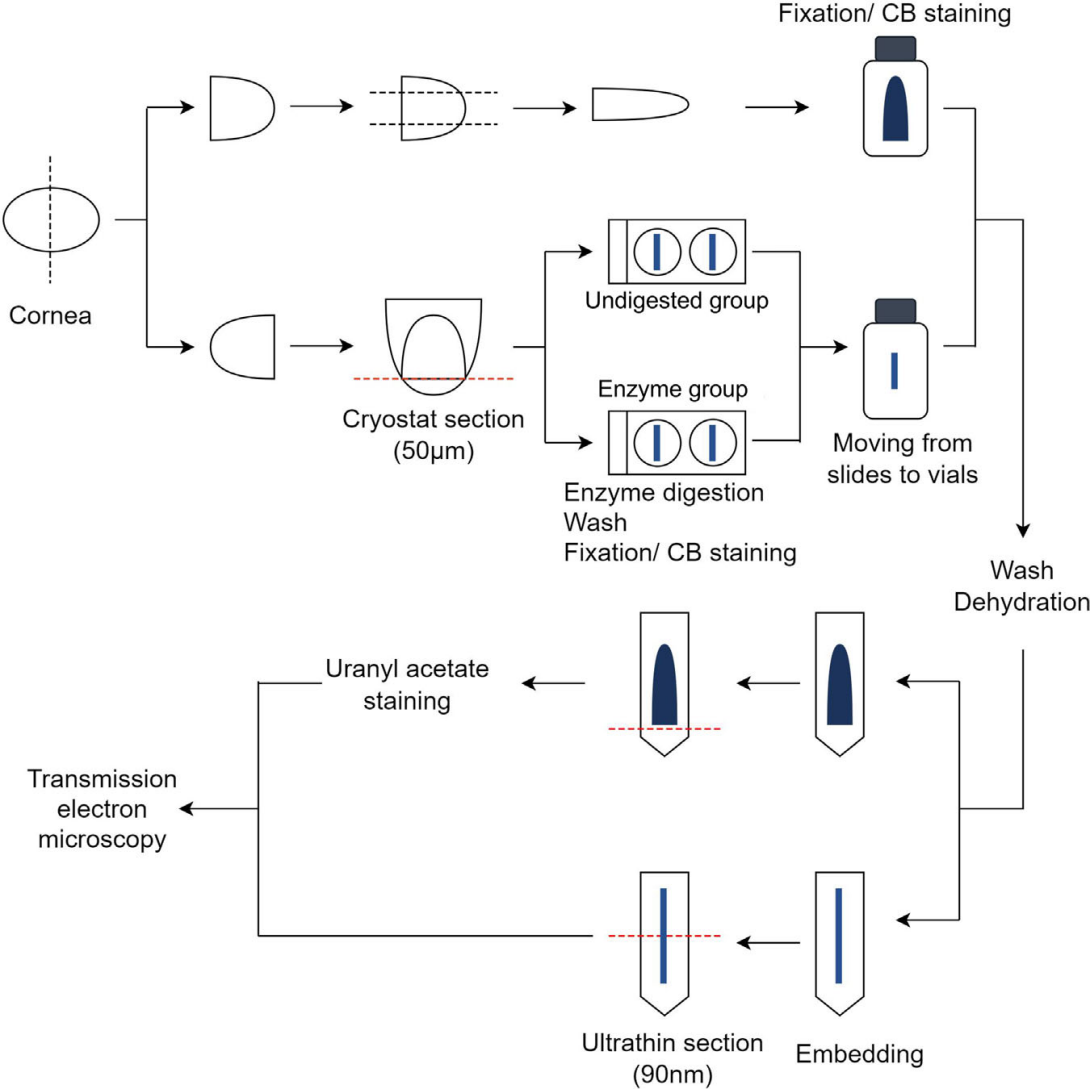
Flowchart for electron microscopy sample preparation. The black dotted lines denote regular scalpel dissection lines, while the red dashed lines indicate cryostat or ultramicrotome sectioning planes. CB, cuprolinic blue.

Frozen rat corneas were thawed and divided into nasal and temporal halves. One-half underwent staining with CB only, and the other one-half were subjected to digestion by chondroitinase ABC (Sigma-Aldrich), followed by CB (British Drug Houses Chemicals Limited, Poole, UK) staining^30^ (Fig. 2). For the one-half of the corneas designated for CB staining only, the middle one-third in the horizontal direction was fixed and stained at room temperature overnight in fixative buffer containing 2.5% glutaraldehyde (Agar Scientific, Essex, UK) and 0.05% CB. The fixative buffer consisted of 2.5 mm sodium acetate (Sigma-Aldrich) (pH 5.7) and 0.1 M MgCl_2_ (Sigma-Aldrich). The other one-half of the corneas were frozen in liquid nitrogen, cryostat-sectioned (Leica CM3050S cryostat, Leica Microsystems [UK] Ltd., Milton Keynes, UK) to a thickness of 50 µm, and collected onto glass slides. Each group of sections was subdivided into two categories: enzyme group and undigested (control) group. The enzyme group was incubated with 2 U/mL chondroitinase ABC in enzyme buffer (pH 8.0) containing 0.02% bovine serum albumin at 37°C for 4 hours. The undigested group was incubated in the same solution but without chondroitinase ABC, under the same conditions. The enzyme buffer comprised 50 mm Tris (Sigma-Aldrich) and 60 mm sodium acetate. After enzyme digestion, the sections were washed in enzyme buffer (pH 8.0), followed by two changes of fixative buffer, and then fixed and stained with 2.5% glutaraldehyde and 0.05% CB in fixative buffer at room temperature overnight. Subsequently, they were transferred from the glass slides to glass vials and processed together with the CB-only specimens. All specimens were sequentially washed three times in fixative buffer and aqueous 0.5% sodium tungstate (Sigma-Aldrich), dehydrated in a graded series of ethanol, and embedded in Araldite CY212 resin (Agar Scientific). Ultrathin sections of 90 nm thickness were obtained using a Reichert UCE ultramicrotome (Reichert, Depew, NY, USA), collected on uncoated G300 copper grids, stained with uranyl acetate or left unstained, and then examined using a JEOL 1010 TEM (JEOL, Tokyo, Japan).

For TEM analysis, a series of images was captured from the anterior, middle, and posterior corneal stroma at various magnifications. Subsequently, five images, all in longitudinal section (LS) and without uranyl acetate stain, were selected each from the anterior, middle, and posterior stroma at an original magnification of ×20,000 and quantification of the PG area was performed using the ROI Manager function in Fiji software version 2.9.0 (National Institutes of Health).

### Immunofluorescence Staining

The entire rat corneas were excised, fixed in 4% paraformaldehyde (Sigma-Aldrich), and embedded in an optimal cutting temperature compound (Leica Microsystems, Wetzlar, Germany) for cryosectioning at an 8 µm thickness. Tissue sections were treated with ice-cold 50 mM ammonium chloride (Sigma-Aldrich), permeabilized with saponin, and blocked using 2% bovine serum albumin and 5% normal goat serum (Thermo Fisher Scientific). Subsequently, they were incubated overnight at 4°C with rabbit polyclonal anti-decorin (Proteintech, Rosemont, IL, USA) at 1:75 dilution or rabbit monoclonal anti-lumican (clone JE11-45; HUABIO, Woburn, MA, USA) at 1:100 dilution. After several washes with 1× PBS (1st BASE, Singapore), the sections were labeled with Red-X-conjugated IgG secondary antibodies (Jackson ImmunoResearch Labs, West Grove, PA, USA) for 1 hour at room temperature. Finally, the sections were mounted with coverslips following nuclei staining with Hoechst 33342 (Thermo Fisher Scientific) and examined under a Zeiss Axioplan 2 microscope (Carl Zeiss, Jena, Germany).

### Statistical Analysis

Statistical analysis was conducted using JMP 17 (SAS Institute, Cary, NC, USA). To compare the SAXS structural parameters of the central cornea between groups, a mixed-effects model was used, with treatment considered as the main factor and animal number as a random factor. Within each cornea, the 13 measured values were treated as repeated measures and nested within each animal. Tukey's comparison test for post hoc analysis was used to identify significant differences between two out of the four groups. Power analysis of the mixed effects model was performed using the Simr package in R (version 4.4.1). Based on 100 simulations, the power for the predictor ‘treatment’ (95% confidence interval) was found to be (96.38–100.0) using the Kenward-Roger test. For quantitative analysis of change in central corneal thickness, haze density and PGs, a one-way ANOVA was applied, followed by Tukey's comparison test. The comparison of clinical haze scoring was analyzed with a Kruskal–Wallis test followed by Dwass–Steel–Critchlow–Fligner pairwise comparisons. The results were presented as mean ± standard deviation and a *P* value of <0.05 was considered statistically significant.

## Results

### Postoperative Corneal Examination

**Figure 3.**
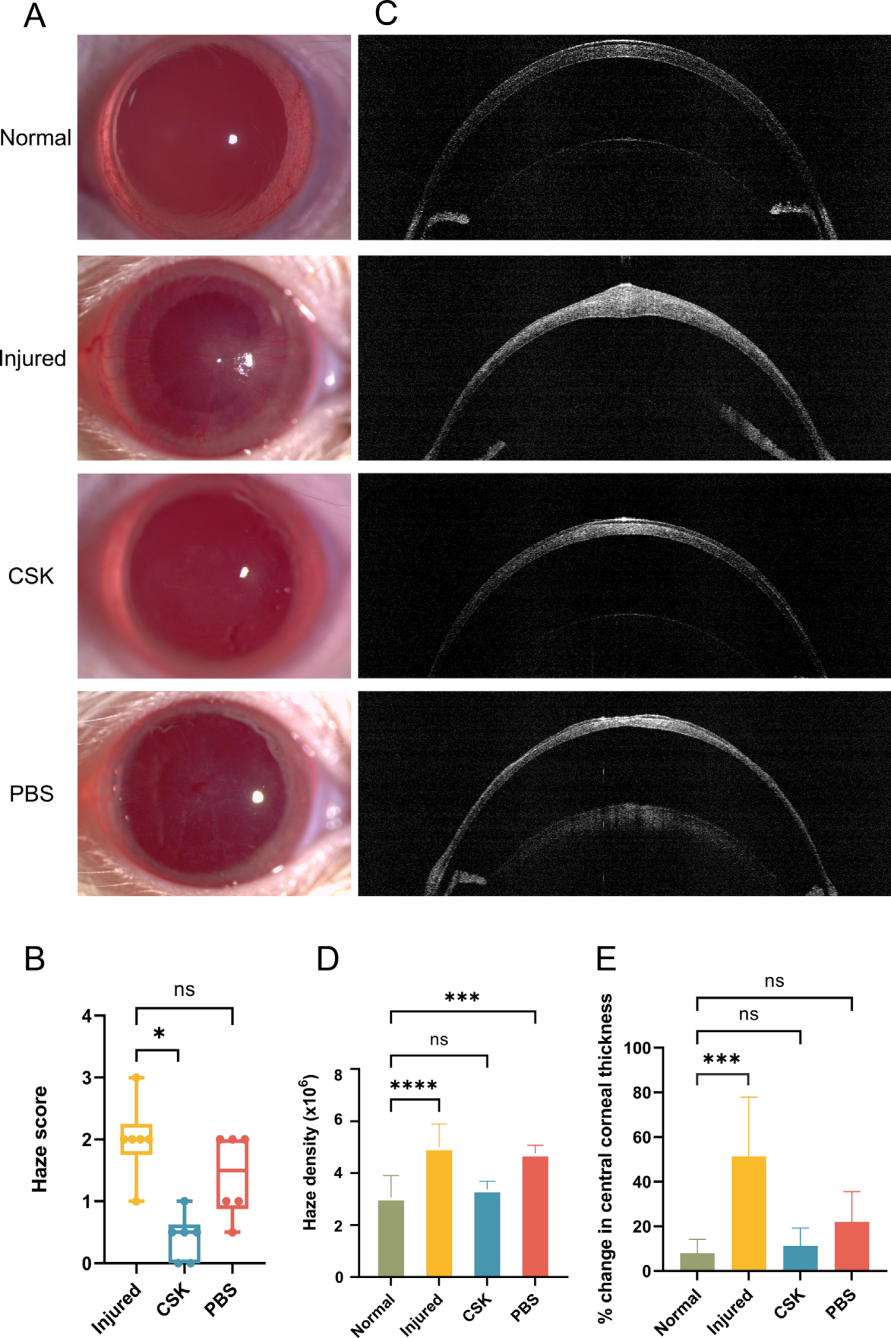
Results of postoperative ophthalmic examination. (**A**) Corneal slit-lamp photographs of normal, injured, CSK, and PBS groups. (**B**) Comparison of slit lamp-based haze score between injured, CSK and PBS groups. (**C**) Corneal AS-OCT images of the normal, injured, CSK, and PBS groups. (**D**) Comparison of AS-OCT–based haze density in the normal, injured, CSK, and PBS groups. (**E**) Comparison of changes in central corneal thickness compared with preoperative in the normal, injured, CSK, and PBS groups. Statistical significance is indicated as follows: *P < 0.05; ***P < 0.001; ****P < 0.0001; ns, *P* ≥ 0.05.

Representative corneal slit lamp images of each group (21 days after CSK injection) are presented in Figure 3A. Central corneal opacity was observed in the injured and PBS groups relative to normal controls, accompanied by the presence of neovascularization originating in the limbus and progressing toward the central cornea. In the CSK group, corneal opacity was notably reduced to levels comparable with normal controls, and there was no visible evidence of neovascularization. The haze score of the injured group was significantly higher than the CSK group (*P* = 0.011), but showed no significant difference from the PBS group (*P* = 0.32) (Fig. 3B). The subjective quantification of the haze with AS-OCT was consistent with the objective slit-lamp–based haze grading. The haze density in the injured (*P* < 0.0001) and PBS (*P* = 0.0003) groups was substantially greater than in the normal group, although there was no statistical difference between the CSK and normal groups (*P* = 0.8086) (Figs. 3C–D). Furthermore, corneal AS-OCT revealed that, compared with the normal group, the change in central cornea in the injured group exhibited marked thickening (*P* = 0.0005). After CSK treatment, the change in corneal thickness returned to near-normal levels (*P* = 0.9503). As shown in Figure 3E, although the change in central corneal thickness of the PBS group exhibited no statistically significant difference compared with that of the normal (*P* = 0.1588) and the CSK (*P* = 0.5008) groups, the change in central corneal thickness in the PBS group was greater than that in the normal and CSK groups. Additionally, the AS-OCT images showed that the corneal surface of the PBS group was not as smooth as the normal and CSK groups (Fig. 3C).

### SAXS

**Figure 4.**
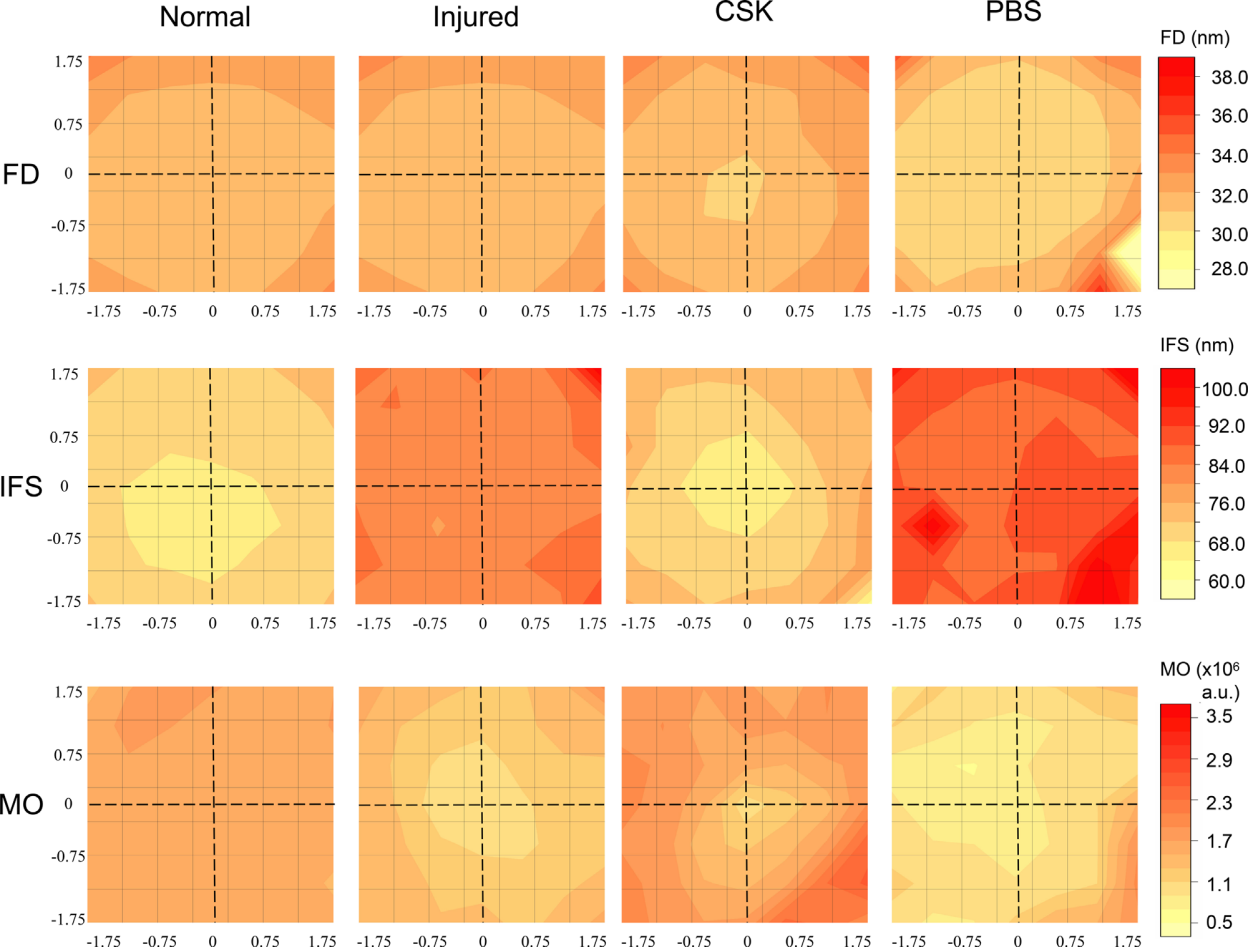
Contour maps of average collagen fibril diameter (FD), interfibrillar spacing (IFS), and matrix order (MO) for the normal, injured, CSK, and PBS groups. The central 3.5 mm × 3.5 mm of each cornea at 0.5 mm × 0.5 mm intervals (7 × 7 data points for each cornea).

SAXS contour maps of the central corneal 3.5 mm × 3.5 mm area, illustrating the structural parameters fibril diameter, interfibrillar spacing and matrix order, are presented in Figure 4. In the normal group, the corneal fibril diameter displayed uniformly lower values centrally and a gradual increase toward the peripheral cornea, aligning with findings in other species^31^^–^^33^ and consistent with requirements for corneal transparency centrally and a merging of the peripheral corneal collagen with the larger diameter fibrils of the sclera.^1^ The injured and PBS groups exhibited a marginal reduction in fibril diameter compared with the normal group. After CSK treatment, the fibril diameter increased without fully reaching the levels observed in the central cornea of the normal group. In line with fibril diameter measurements, collagen interfibrillar spacing in the normal group gradually increased from the central to the peripheral cornea, again consistent with findings in other species.^31^^,^^32^ Corneal injury resulted in increased interfibrillar spacing, with CSK treatment restoring values to near normal levels, although the PBS group exhibited significantly higher interfibrillar spacing compared with the normal group. Matrix order represents the degree of regularity in the spatial organization of collagen fibrils. In comparison with the normal group, the injured and PBS groups exhibited a marked decrease in matrix order across the central 3.5 mm × 3.5 mm corneal region, indicative of more disorganized collagen fibrils. Subsequent CSK treatment resulted in a near-complete recovery, with matrix order reaching levels similar to the normal group.

**Figure 5.**
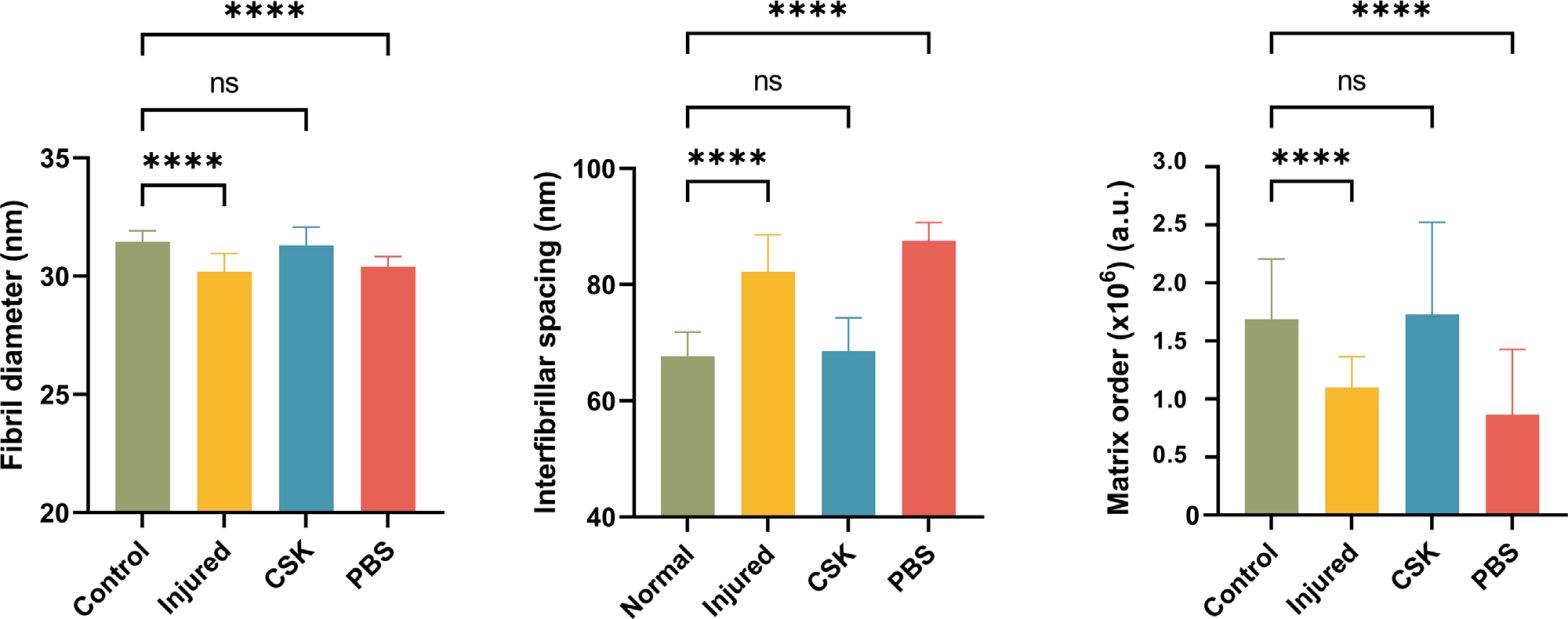
Comparison of average collagen fibril diameter, interfibrillar spacing, and matrix order in the normal, injured, CSK, and PBS groups within the central 2 mm × 2 mm area (13 data points per cornea, *n* = 156 for the normal group, *n* = 65 for the injured and PBS groups, and *n* = 78 for the CSK group). Statistical significance is indicated as follows: ****P < 0.0001; ns, *P* ≥ 0.05 as compared with the normal group.

To further quantify stromal ultrastructural parameters, a central 2 mm × 2 mm region of each cornea (13 values per cornea), in which parameters are uniform in the normal rat cornea, was selected for statistical analysis (Fig. 5). Fibril diameter in the injured (*P* < 0.0001) and PBS (*P* < 0.0001) groups exhibited a statistically significant decrease compared with the normal group, albeit the absolute extent of reduction was relatively modest at approximately 2 nm. Conversely, there were no significant differences in fibril diameter between the CSK and normal control groups (*P* = 0.1139). Corneal injury led to an increase in the interfibrillar spacing of the corneal stroma (*P* < 0.0001), and the PBS group (*P* < 0.0001) exhibited a similar increase, with both significantly higher than the normal group. Subsequent CSK injection successfully restored interfibrillar spacing to levels comparable with the normal group (*P* = 0.5879). In contrast with the normal group, the matrix order in the injured (*P* < 0.0001) and PBS (*P* < 0.0001) groups saw a substantial and significant decrease, amounting to approximately 50%, a decrease that was largely reversed by subsequent CSK injection (*P* = 0.9999).

### CB-TEM

**Figure 6.**
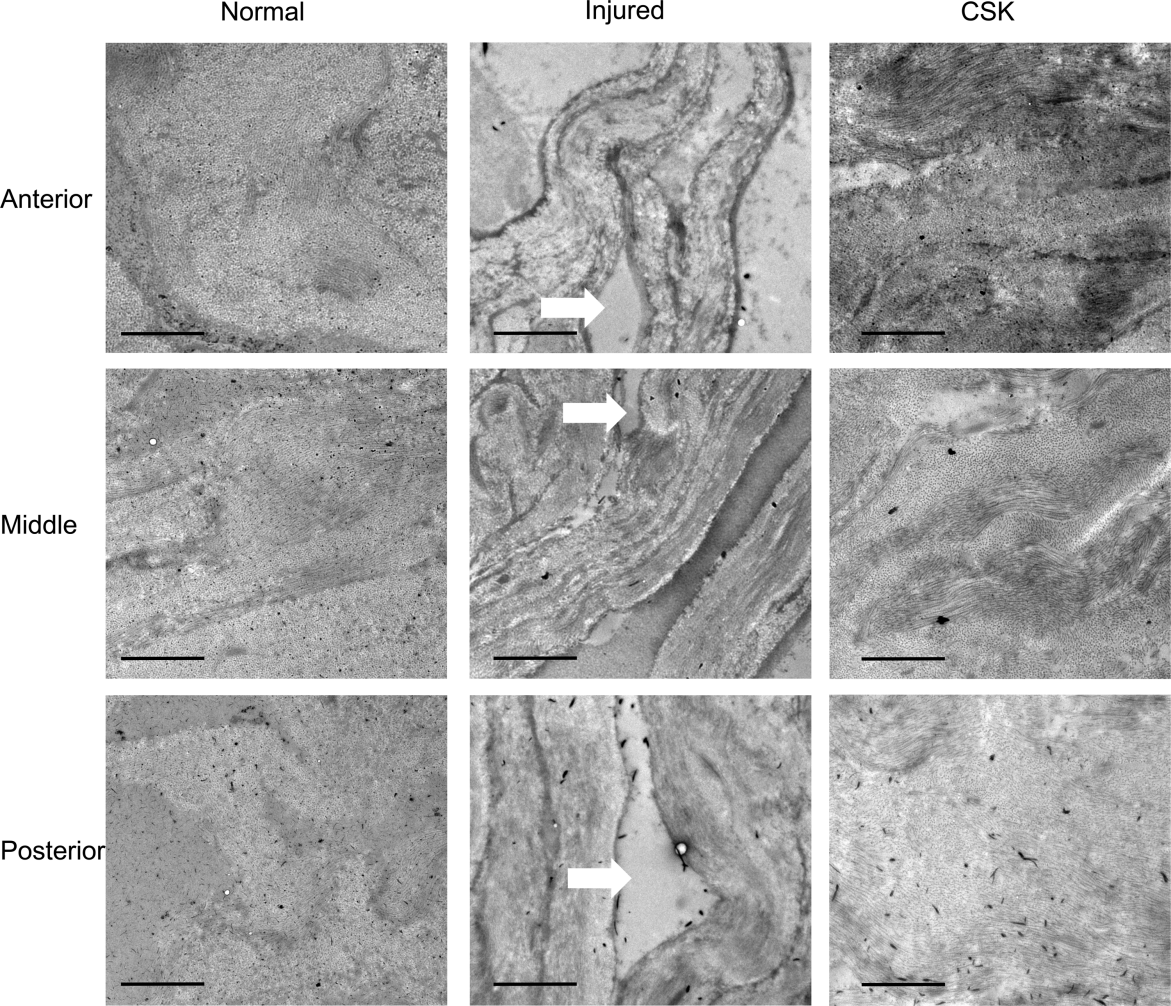
TEM images with CB staining in the anterior, middle, and posterior stroma of the normal, injured, and CSK groups. *Arrows* denote collagen-free lakes in injured group. (*Scale bar*, 2 µm; original magnification ×1,500.)

At an original magnification of 1500× (Fig. 6), CB-TEM images of the anterior, middle, and posterior corneal stroma for the normal group revealed tightly packed, undulating, and interwoven collagen lamellae. In contrast, the injured group exhibited considerable disruption with numerous large collagen-free lake regions visible. CSK injection restored the lamellar organization to a state comparable with the normal group.

**Figure 7.**
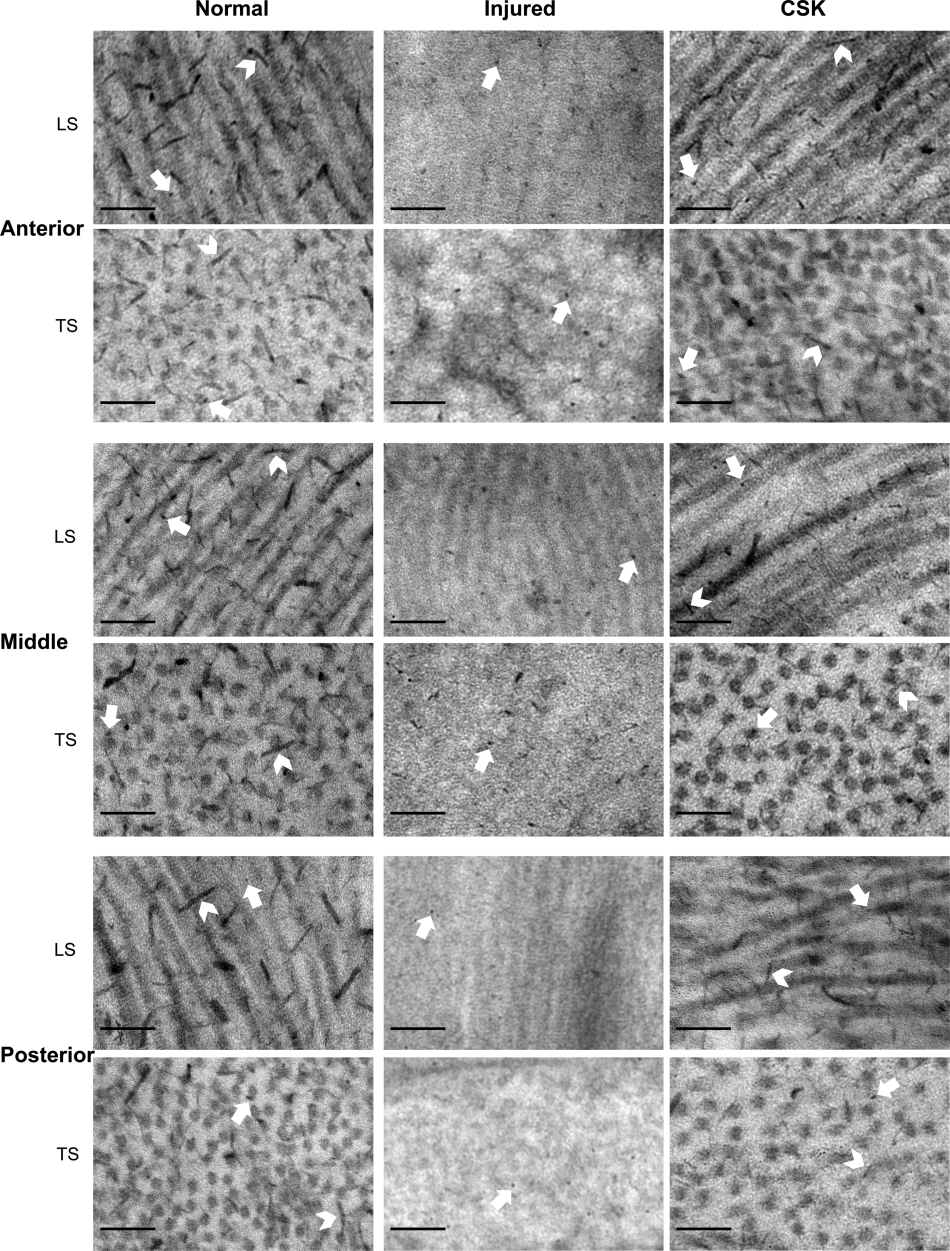
TEM images with CB staining of anterior, middle, and posterior corneal stroma of normal, injured, and CSK groups. TS, transverse section. *Arrowheads*, filamentous PGs; *Arrow*, dot-like PGs. (*Scale bar*, 100 nm; original magnification, ×12,000.)

At a higher original magnification of ×12,000 (Fig. 7), the normal group displayed parallel alignment of collagen fibrils in LS. In transverse section, collagen fibrils demonstrated largely uniform diameters and regular spacing. CB staining allowed visualization of PGs. In LS, PGs appeared as electron-dense filaments or dots, associated with collagen fibrils. Most of the dot-like PGs were localized on the fibril surface, with few dispersed between fibrils. Filamentous PGs of varying lengths ran both parallel to fibrils and transversely, the latter bridging one or more adjacent fibrils. These observations were also confirmed by the association of PGs and collagen in transverse section. Although the CSK group exhibited similar collagen fibrillar arrangement and PG distribution to the normal group, the injured group displayed, in contrast, poorly defined collagen fibrils and lower numbers of PGs that were sparsely distributed and mostly presented as dots or short rods, with minimal filamentous PGs visible.

### CB-TEM With Chondroitinase ABC Digestion

**Figure 8.**
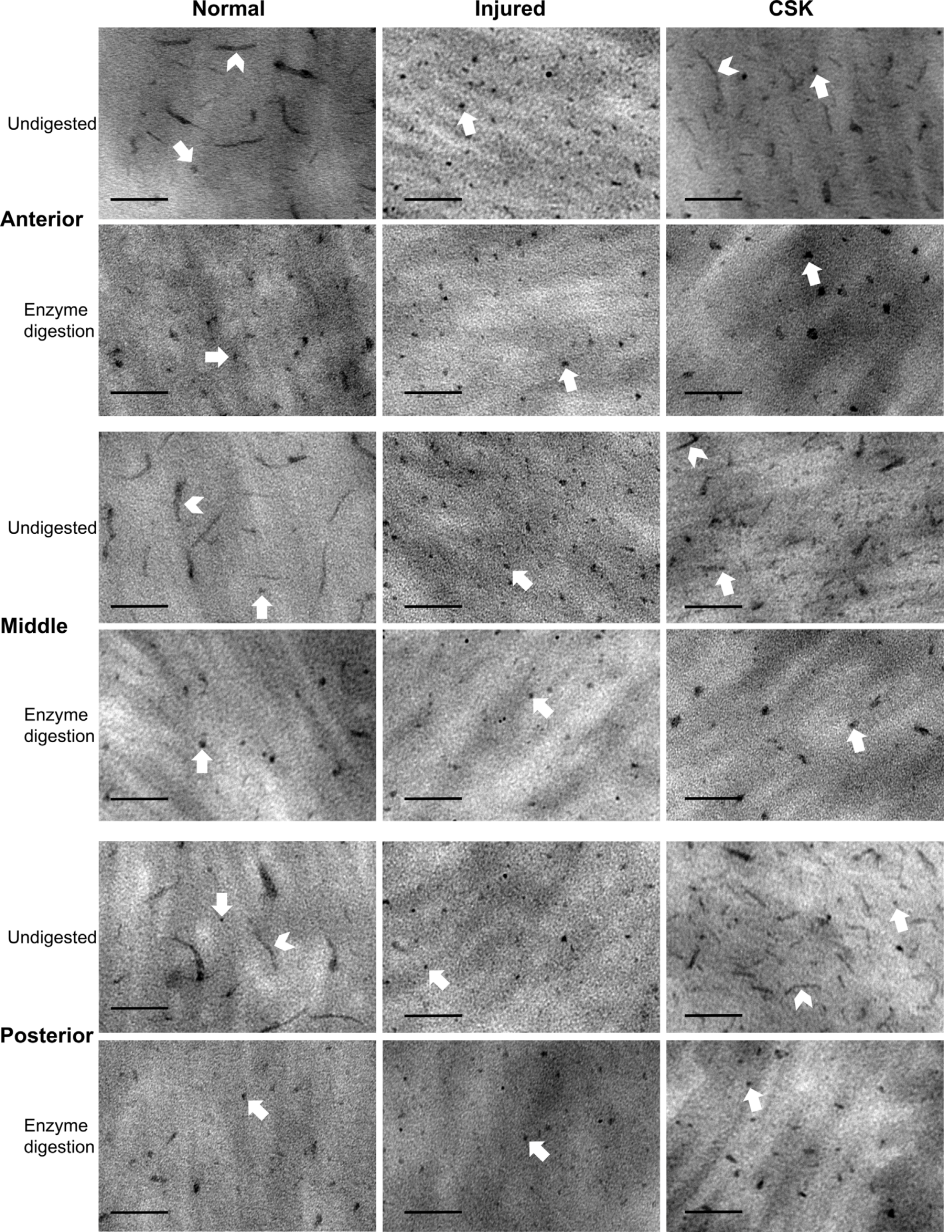
TEM images of chondroitinase ABC enzyme-digested specimens and undigested controls in the anterior, middle, and posterior stroma of normal, injured, and CSK groups. *Arrowheads*, filamentous PGs; *Arrow*, dot-like PGs. (LS without uranyl acetate staining.) (*Scale* *bar*, 100 nm; original magnification, ×20,000.)

CB-TEM images (original magnification, ×20,000) of the normal, injured, and CSK groups with chondroitinase ABC predigestion are compared with respective undigested controls in Figure 8. Uranyl acetate staining was omitted to reduce the intensity of collagen and facilitate clearer observation of PGs stained by CB. In the undigested control of the normal group, both dot-like and filamentous electron-dense PG structures were evident. In contrast, the undigested control of the injured group displayed only dot-like PGs, which were largely unaffected by enzyme digestion. The undigested control of the CSK group indicated partial restoration of normal PG distribution and appearance, with filamentous structures largely restored, but with a shorter maximum length compared with the normal group.

**Figure 9.**
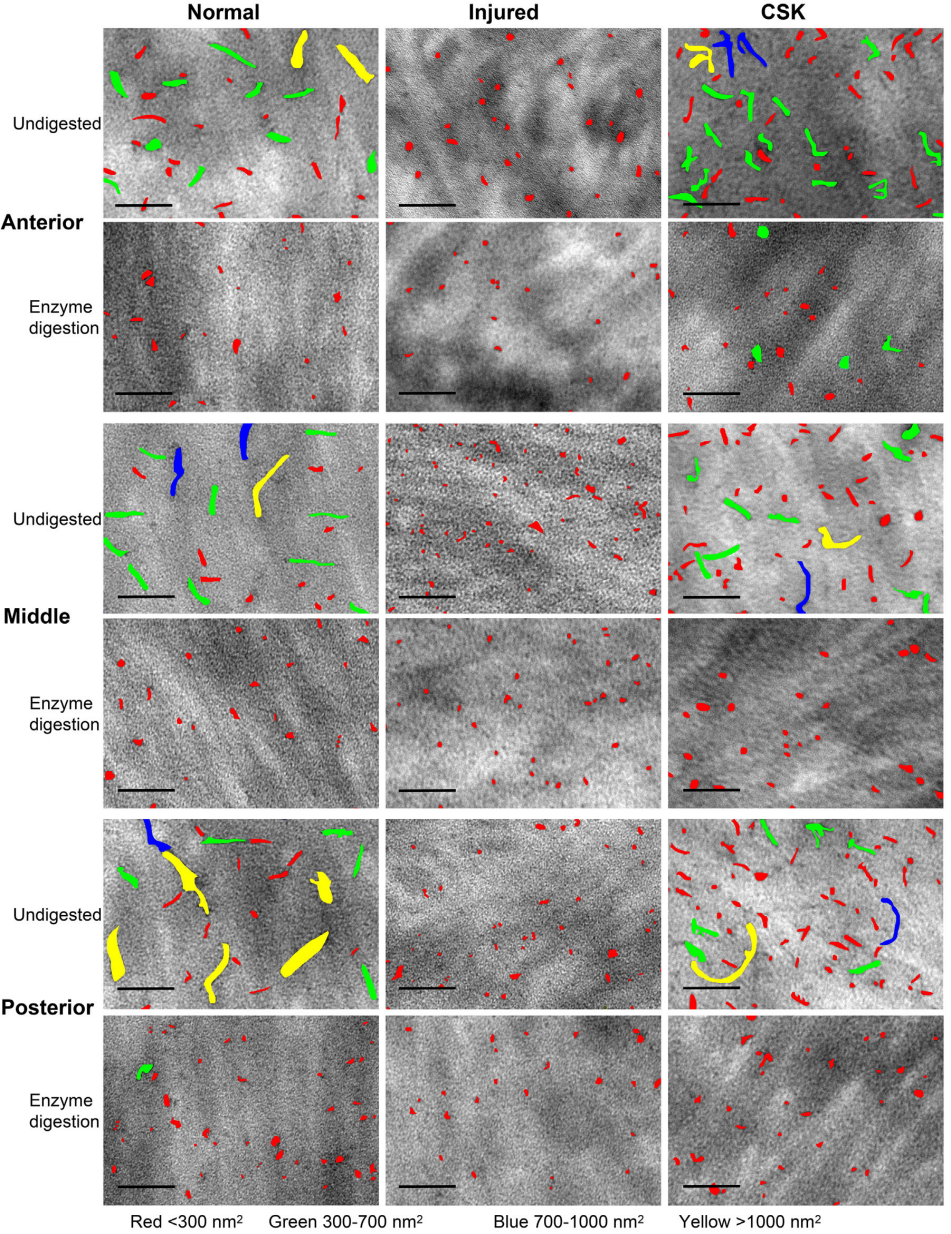
Manually marked PGs in the anterior, middle, and posterior stroma of the normal, injured, and CSK groups before and after chondroitinase ABC enzyme digestion using the ROI Manager function in Fiji software. Different colors represent the varying sizes of individual PG areas: *red*, <300 nm²; *green*, 300 to 700 nm²; *blue*, 700 to 1000 nm²; *yellow*, >1000 nm². The samples were not stained with uranyl acetate and are presented as a LS at an original magnification of ×20,000. (*Scale bar*, 100 nm.)

**Figure 10.**
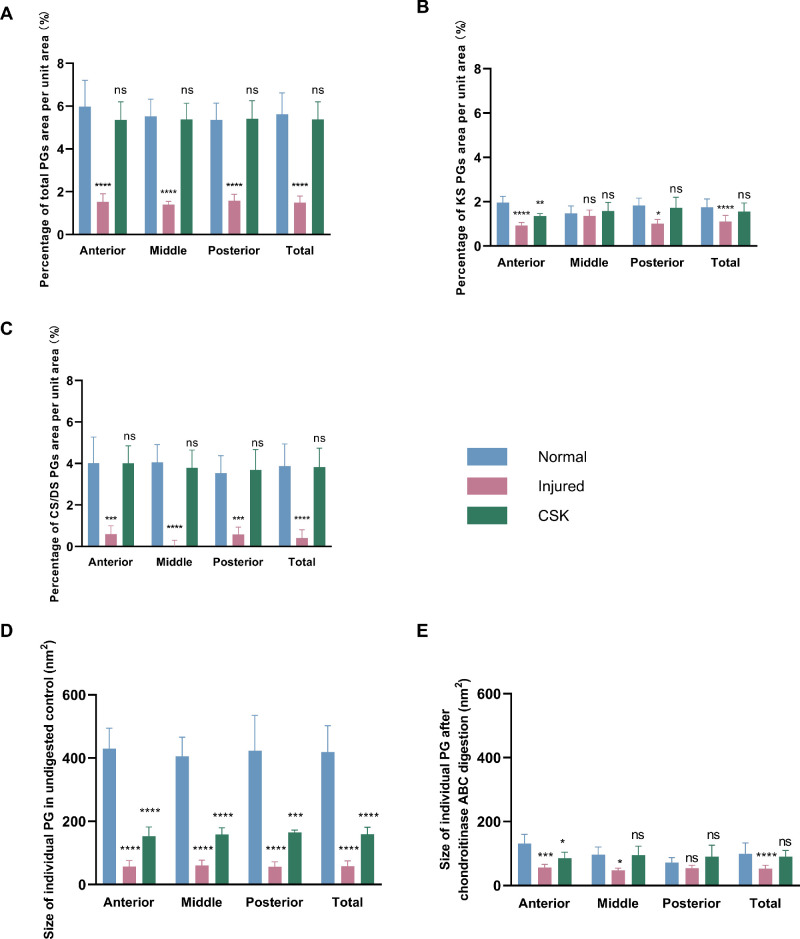
Quantitative analysis of CB-TEM. Comparison of the percentage of the total area of PGs (**A**), KS PGs (**B**), and CS/DS PGs (**C**) in the unit area in the anterior, middle, posterior, and entire corneal stroma of the normal, injured, and CSK groups. Comparison of average size of individual PGs in undigested control (**D**) and after enzyme digestion (**E**) in the anterior, middle, posterior, and entire corneal stroma of normal, injured, and CSK groups. Statistical significance compared with the corresponding normal group is indicated as follows: *P < 0.05; **P < 0.01; ***P < 0.001; ****P < 0.0001; ns, *P* ≥ 0.05.

An interpretation of the PG morphology described herein can be made with reference to previously published work with critical electrolyte staining in rat cornea. Unlike some other rodent species, including mice (which do not contain significant amounts of sulphated KS), the adult rat cornea is abundant with both CS/DS- and KS-rich PGs,^34^^,^^35^ of which the former, larger GAG structures can be readily and selectively removed using chondroitinase ABC enzyme to leave only the smaller KS-rich populations.^35^ Using this as our basis, a quantitative analysis of LS images (Fig. 9) was conducted to evaluate the relative percentage of KS (attributed to dot-like structures) and CS/DS (attributed to larger filamentous structures) PG staining per unit area, across the anterior, middle, posterior stromal regions and overall (Figs. 10A–C). In the undigested control, a significant decrease (*P* < 0.0001) in the percentage of total PG staining per unit area was observed in the injured group compared with the normal group in all corneal stromal regions. After enzymatic digestion, both the percentage of KS-attributable (retained CB-stained structures) and CS/DS-attributable (removed CB-stained structures) PGs per unit area were found to be significantly decreased in the injured group compared with the normal group. This effect was particularly pronounced for the filamentous CS/DS PGs. In contrast, the percentage of total PG staining per unit area in the CSK group, both before and after digestion, did not significantly differ from that in the normal group, reflecting a recovery in PG populations.

Quantitative analysis of the average size of PGs in the anterior, middle, posterior, and total corneal stroma before digestion (Fig. 10D) revealed that the injured group exhibited a significant decrease in average PG size compared with the normal group (*P* < 0.0001) of approximately 85%. Although subsequent CSK treatment increased the average size of PGs compared with the injured group, it remained approximately 60% lower than that of the normal group. After enzymatic digestion of CS/DS GAG chains (Fig. 10E), the average size of PGs in the injured group was significantly lower than the normal group by approximately 50%, but this decrease was largely reversed by subsequent CSK treatment, reaching 90% of normal levels.

### Immunofluorescence Staining

**Figure 11.**
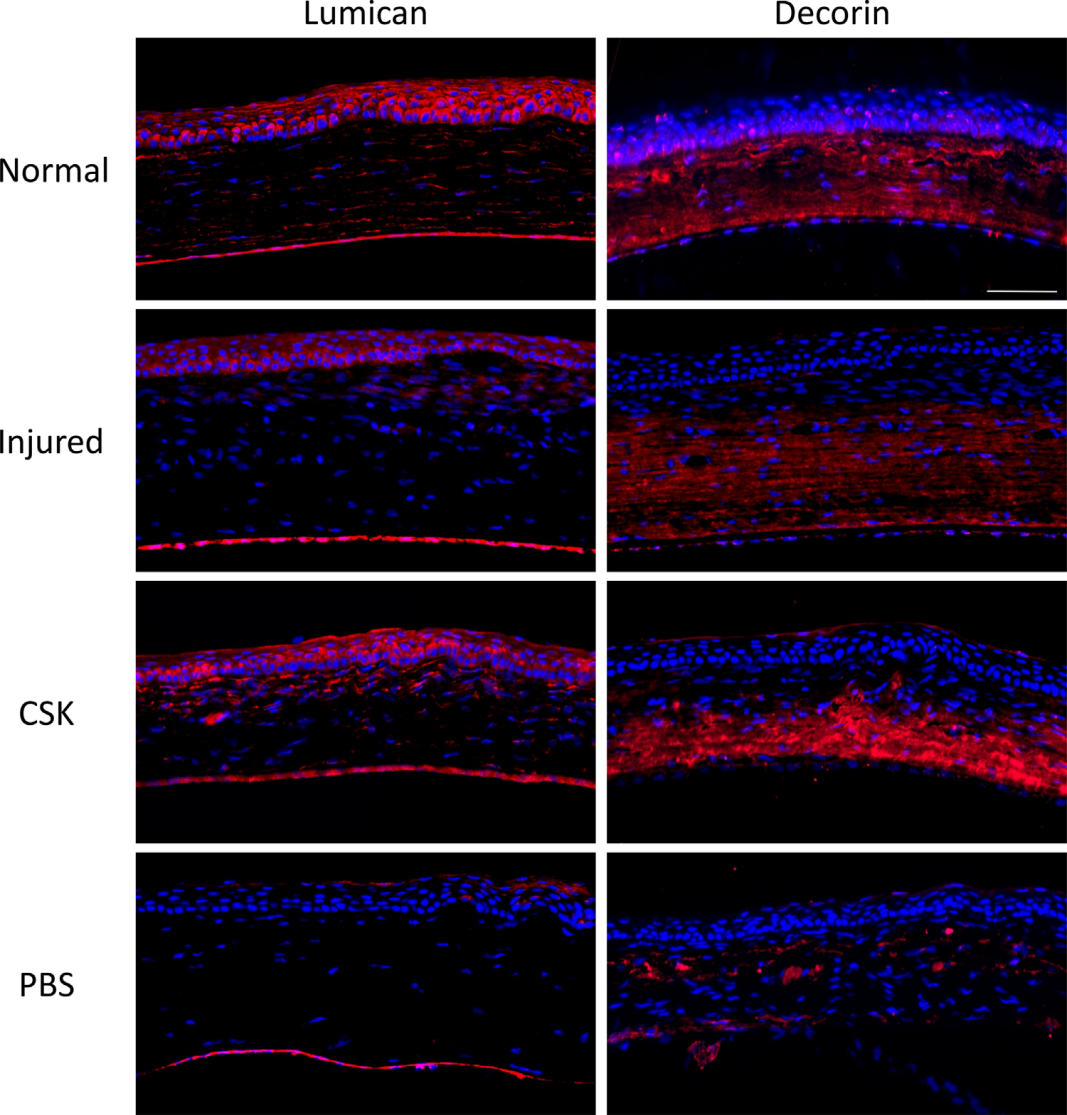
Immunofluorescence staining images of lumican and decorin for the normal, injured, CSK, and PBS groups. (*Scale* *bar*, 100 µm.)

The fluorescence staining images of lumican and decorin are shown in Figure 11. In the normal group, lumican was distributed throughout the entire corneal epithelium and stroma. In the injured group, lumican expression was limited to the corneal epithelium and some part of superficial stroma near the epithelium. After CSK treatment, lumican redistributed across the full thickness of the corneal stroma. In contrast, the PBS group showed almost no lumican expression across the whole cornea. Decorin was evenly distributed throughout the normal corneal stroma. In the injured group, although decorin was expressed in the noninjured areas, its intensity was lower compared with the normal group. In the CSK treatment group, the intensity of decorin was significantly higher than the injured group, and some expression was also observed in the injured areas. In the PBS group, there was only scattered decorin expression in the corneal stroma.

## Discussion

We have previously conducted detailed studies^16^^,^^18^^,^^20^ regarding the mechanisms by which CSK influences corneal wound healing and stromal restoration. After corneal injury, the transformation of native CSKs into fibroblasts triggers inflammation and the release of proliferative cytokines, leading to collagen disorganization and an abnormal extracelluar matrix. In contrast, injected quiescent CSKs can perform their inherent functions effectively, including the secretion of molecular extracelluar matrix components and collagen fibril reorganization. Given that the arrangement of collagen and the distribution of PGs are decisive factors for corneal transparency, the current study was designed to assess the ultrastructural effects of CSK treatment on stromal extracelluar matrix components, specifically collagen and PGs. We found using SAXS that the recovery of corneal transparency by CSK treatment was associated with the restoration of collagen fibril diameter, interfibrillar spacing, and matrix order, to normal or near-normal levels. Furthermore, by using chondroitinase ABC enzyme digestion before CB-TEM, we were able to study the impact of injury and CSK treatment on the stromal PGs. CSK-treated corneas had both CS/DS and KS PG populations restored to levels comparable with those of normal controls, although the average size of PGs in the CSK-treated group remained relatively smaller. These effects were, in major part, attributable to injury-related loss and the subsequent therapeutic recovery of filamentous CS/DS PGs.

SAXS has been used extensively to quantify the ex-vivo nanoscale organization of corneal stromal collagen fibrils.^36^^–^^38^ The current results (Figs. 4, 5) indicated that irrPTK injury led to significant disruption of collagen fibrillar size, spacing, and order. The inflammatory cascade in response to stromal injury activates the release of proinflammatory cytokines, which promotes vasodilation and increases vascular permeability, ultimately leading to tissue edema.^6^^,^^39^ The measured increase in interfibrillar spacing in injured corneas reported here using SAXS thus aligns with an inflammatory-type injury response, and this result was supported by our observation, via AS-OCT, of corneal thickening (Figs. 3C, 3E) and, via TEM, of collagen-free stromal lakes (Fig. 6), which likely contributes to the overall reduced levels of collagen matrix order as detected by x-rays (which penetrate and average the full stromal thickness). It is worth noting that the change in central corneal thickness of the PBS group was not as large as that of the injured group (Fig. 3E). The superficial layer of the stroma was likely removed at the needle's entry point, an effect not experienced by the injured group. This factor may have affected the postoperative central corneal thickness measurements, leading to an underestimation in the PBS group.

Interestingly, our SAXS analysis also demonstrated a slight reduction (approximately 2 nm) in fibril diameter after injury (Figs. 4, 5). Corneal injury and healing are complex processes that vary markedly depending on the injury type.^40^^,^^41^ Histological studies of excimer laser application to the cornea suggest that localized, transient increases in stromal temperature may induce collagen contraction, which could explain the observed decrease in fibril diameter.^42^ This scheme is distinct from corneal injury models that induce more established scarring (e.g., penetrating wounds), where collagen fibril diameters are typically enlarged in the generation of persistent fibrotic matrix.^32^^,^^43^ CSK treatment after laser-induced injury restored fibril diameter, interfibrillar spacing, and matrix order to levels approaching those seen in the normal group (Figs. 4, 5), observations that confirm our previously reported ultrastructural findings.^20^ Moreover, the similarity of TEM images between the CSK-treated group and the normal group, characterized by tightly packed stromal collagen lamellae (Figs. 6, 7), substantiates our SAXS findings, and is concordant with the therapeutic recovery of corneal transparency and thickness observed in vivo via slit-lamp and AS-OCT examination (Fig. 3). This result further aligns with our previous findings that intrastromal injection of human CSKs can restore corneal transparency in injured corneas without triggering significant additional immune or inflammatory responses.^16^^,^^20^

We also used CB critical electrolyte staining, in combination with TEM, to observe the impact of irrPTK-induced injury and subsequent CSK treatment on the distribution of stromal PGs.^44^^–^^48^ We selectively digested CS/DS GAGs with chondroitinase ABC before CB staining to observe changes in KS PGs,^5^ visible as electron-dense dots or short rods. We then compared the results with the undigested samples to assess alterations in CS/DS PGs, visible as larger filamentous structures. CSK injection was found to largely reverse the injury-induced decrease in both KS and CS/DS PG populations, as measured by the corresponding CB stain occupancy per unit area of the stroma under TEM, with the loss and restoration of CS/DS structures being particularly marked (Figs. 10A–C). Given the central role that small leucine-rich proteoglycans play in the maintenance of corneal transparency and the promotion of tissue repair,^6^ we contend that the restoration of CS/DS and KS PG distribution is an important contributing factor in the CSK-enhanced recovery of corneal transparency after laser injury reported herein and previously.^16^^,^^20^

Specifically, the primary CS/DS PG decorin, in addition to its well-documented role in regulating collagen fibrillogenesis and organization,^49^ can neutralize the prodifferentiative effects of TGF-b to promote corneal wound healing, inhibit neovascularization, and suppress fibrotic scarring,^50^^,^^51^ with biglycan compensating in the absence of decorin when necessary.^6^ Among the KS PGs, lumican has a major role in modulating corneal transparency.^4^^,^^52^^,^^53^ Additionally, lumican has been shown to stimulate epithelial cell migration and adhesion and promote the recruitment of macrophages and polymorphonuclear neutrophils, thereby expediting corneal wound healing.^54^ Our immunofluorescence staining images (Fig. 11) showed that CSK treatment restored the decrease in lumican and decorin expressions caused by laser injury. Combined with our previously published immunofluorescence staining results of keratocan,^20^ although a direct comparison cannot be made owing to differences in experimental principles and methods, these findings support our TEM results, indicating that CSK can restore CS/DS and KS PGs in injured corneas. A caveat here is that variations in the type and severity of corneal injury complicate the interpretation of the present results when drawing comparisons with the published literature. Moreover, the timing of both after injury and treatment stage observations is key. Indeed, in the current study we note that the average size of individual PGs remained significantly lower than that of the normal group (Fig. 10D), with the number of PGs (Fig. 8) increasing such that the total staining of PGs per unit area was restored to normal levels (Fig. 10A). This finding indicates that, although the overall PG content was normalized, the smaller average size of the PGs suggests that a longer recovery period may be required for full restoration. It is possible that, on average, GAG chain length/number and/or sulfation levels of individual CS/DS PGs may not have fully reached quiescent levels at the 3-week recovery timepoint investigated here following a single cell injection.

The current work is subject to limitations. First, the findings are based on xenograft models, where human cells were injected into rat corneal stroma, which may limit the direct applicability to human therapeutics. However, similar promising results have been observed in other mammalian models,^55^ and together with this study, they represent an important step forward toward possible clinical trials. Second, SAXS does not provide direct imaging of the corneal structure but rather produces a scattering pattern (Fourier transform) that averages the whole corneal thickness. TEM, in contrast, offers direct high-resolution imaging, albeit with a restricted field of view, and thereby serves as a complementary technique to SAXS. Last, further ultrastructural studies that extend the observation time and assess the possible impact of repeated CSK injections are warranted.^16^

In conclusion, we investigated the effects of intrastromal injection of quiescent CSKs on corneal stromal collagen fibril arrangement and PG distribution in a rat model of laser-induced corneal injury. The observed restoration of corneal transparency and stromal matrix ultrastructure endorses the therapeutic potential of CSK treatment. Our previously published preliminary results^20^ showing that CSK decreased scarring in a chronic corneal opacity model, albeit with an attenuated efficacy compared with the acute opacity model, suggests the potential of CSK in treating advanced-stage corneal opacity. If there is associated cornea thinning or very severe thinning, cell injection could be combined with some stromal expansion, for example, recombinant cornea, bioengineered cornea, or liquid cornea, and we are looking at this option now. In clinical practice, the treatment of corneal injuries depends not only on the type and stage of the injury, but also the clinical setting and local infrastructure. For example, in higher income countries treatment can typically be initiated within 1 week. This prompt response is enabled by advanced health care systems, efficient transportation and shipping networks, and easier access to tertiary-level health care. In contrast, in low-income countries with less developed infrastructure and a scarcity of corneal specialists, treatment delays are common. Our previous study^20^ demonstrated that delayed presentation of the scar affected the efficacy of the CSK therapy. Nonetheless, optimizing outcomes for delayed CSK treatment remains crucial and will be the focus of a separate study by our group. Findings from the current study underscore the importance of early CSK intervention, which was shown to effectively resolve corneal haze. This outcome was achieved through the restoration of the collagen matrix architecture and PG composition to a state closely resembling the native corneal stroma. Nevertheless, the focus of the present study is on early stage corneal injuries, and further research is required to facilitate the possible integration of such approaches into clinically deliverable strategies for the treatment of corneal disorders and injury, particularly for more severe and/or established corneal scarring, for example, fibrotic wounds and after infection.
